# Myasthenia Gravis—An Analysis of Multimodal Evoked Potentials

**DOI:** 10.3390/brainsci11081057

**Published:** 2021-08-11

**Authors:** Edyta Dziadkowiak, Marta Waliszewska-Prosół, Małgorzata Wieczorek, Joanna Bladowska, Sławomir Budrewicz, Maria Ejma

**Affiliations:** 1 Department of Neurology, Wroclaw Medical University, 50-556 Wroclaw, Poland; edbkowal@gmail.com (E.D.); s.budrewicz@wp.pl (S.B.); mejma@interia.pl (M.E.); 2 Faculty of Earth Sciences and Environmental Management, University of Wroclaw, 50-137 Wroclaw, Poland; malgorzata.wieczorek@uwr.edu.pl; 3 Department of General Radiology, Interventional Radiology and Neuroradiology, Wroclaw Medical University, 50-556 Wroclaw, Poland; asia.bladowska@gmail.com

**Keywords:** myasthenia gravis, visual evoked potentials, brainstem auditory evoked potentials, somatosensory evoked potentials

## Abstract

Introduction: The aim of this study is a comprehensive analysis of the parameters of exogenous evoked potentials (visual, brainstem auditory, and somatosensory) in patients with myasthenia gravis (MG), a prototype of both neuromuscular junction disease and autoimmune disease. The study also seeks to isolate electrophysiological changes that may indicate disorders within the central and/or peripheral nervous system. Methods: A total of forty-two consecutive patients with myasthenia gravis (24 women, 18 men) were included in the study. All of the patients underwent EP examination. MR images were also analyzed. Results: In the group of MG patients, the latency of P100 (113.9 ± 13.9; *p* < 0.0001) VEP, wave III (3.92 ± 0.29; *p* = 0.015), wave V (5.93 ± 0.32; <0.0001), interlatency III–V (2.00 ± 0.12; *p* < 0.0001), interlatency I–V (4.20 ± 0.28; *p* < 0.001) BAEP, and all components of SEP (N9, P10, N13, P16, N20, P22) were significantly longer. Mean wave I and V amplitude BAEP were relatively lower. Conclusions: The results of the study suggest the presence of disturbances in the bioelectric activities of the central and peripheral nervous system in MG patients.

## 1. Introduction

Myasthenia gravis (MG) is an autoimmune disease of the postsynaptic part of the neuromuscular junction. The severity of symptoms ranges from mild ocular symptoms to the life-threatening condition of myasthenic crisis. The estimated prevalence of myasthenia gravis is 15–179 per million people. The first symptoms of myasthenia gravis usually appear in women under 40 years of age and in men after the age of 50 [1,2].

In myasthenia gravis, pathogenic anti-acetylcholine receptor antibodies (AChRAb) are always present. Almost two-thirds of patients with myasthenia gravis of early onset, i.e., beginning before the age of 50, have persistent thymus. AChRAb is found in approximately 85% of patients with nonthymomatous myasthenia gravis; the remaining 15% are seronegative. The prevalence and titer of AChRAb are lower in ocular myasthenia gravis but do not clearly correlate with the severity of symptoms in generalized myasthenia gravis. The antibody titer is not an indicator of improvement after treatment. In seronegative myasthenia gravis, antibodies to muscle-specific tyrosine kinase (MuSK) are present in a certain percentage of cases. This form is called MuSK-positive myasthenia gravis and is characterized by certain clinical peculiarities and is less amenable to treatment. The third group is called “double seronegative” myasthenia gravis and is without both AChR-Abs and anti-MuSK antibodies. Clinically, it does not differ from seropositive myasthenia gravis; AChR-Abs may be present in this group but at very low titers and with low affinity for receptors, so they cannot be detected. Antibodies against muscle proteins are also important: against titin (a protein at striatum I responsible for muscle elasticity), ryanodine (an anti-RyR, sarcoplasmic reticulum calcium channel protein) and ropsin (an anti-RAPSN, intracellular protein), andlow-density lipoprotein-related protein 4 (Lrp4) [1,2,3].

Myasthenia gravis may coexist with other autoimmune diseases such as thyroid disease, rheumatoid arthritis, lupus erythematosus, multiple sclerosis, pemphigus, and psoriasis [4].

The primary diagnostic test in myasthenia gravis is the electromyographic stimulation test and single muscle fiber electromyography, which is the most sensitive method for evaluating the abnormalities of neuromuscular transmission. Evoked potentials (EP) are another electrophysiological method that is not routinely used in myasthenia gravis but that provides information on the function of the peripheral and central nervous system. Evoked potentials allow the determination of changes in the bioelectrical activity of the nervous system caused by the irritation of afferent pathways. We distinguish between exogenous potentials, which are a direct response to presented sensory stimuli, and endogenous potentials, which are temporarily related to cognitive processes or motor activity planning. Exogenous potentials are most often obtained through the stimulation of the visual, auditory, and somatosensory pathways and the further stimulation of the relevant cortical field representations. Exogenous potentials depend on the physical properties of the stimulating stimulus; their latencies are shorter than those of the endogenous potentials, and they reach their maximum amplitude over the primary cortex that is adequate for the given stimulus modality. In the case of VEPs, the highest evoked response is registered over the visual cortex, and in the case of SEPs, this is over the parietal cortex on the side opposite to the stimulated nerve. BAEPs are generated in the nerve VIII and brainstem structures. SEPs are a sensitive indicator of subclinical damage to nervous system structures in the course of various diseases, but they are not specific for their etiology. It has also been shown that the parameters of evoked responses and the dynamics of their changes may correspond to the clinical course of the disease [5].

The aim of this study is a comprehensive analysis of the parameters of exogenous evoked potentials (visual—VEP, brainstem auditory—BAEP, somatosensory—SEP) in patients with myasthenia gravis, a prototype of both neuromuscular junction disease and autoimmune disease. The study also seeks to isolate electrophysiological changes that may indicate disorders within the central and/or peripheral nervous system.

## 2. Materials

The study group consisted of 42 patients (including 24 women and 18 men). The mean age of the patients was 56.5 (SD = 19.1; women 52.3, SD = 20; and men 62.1, SD = 16.7). The control group included 50 healthy volunteers (43 females and 7 males, average age 46.24; 46.53 for females and 44.43 for males).

A total of eighteen patients had ocular forms of myasthenia gravis, and 24 patients had generalized forms. The disease duration ranged from 1 to 35 years. In 12 patients, other autoimmune diseases coexisted—Hashimoto’s disease (*n* = 6) and rheumatoid arthritis (*n* = 2). Hypertension was diagnosed in 8 patients, type 2 diabetes in 9, hypothyroidism in 4, and hyperthyroidism in 1. Acetylcholine receptor antibodies were found in 38 patients, and anti-MuSK antibodies were found in 4 patients.

In a neurological examination of the patients, apart from typical symptoms of muscle fatigue, there were no signs of focal or diffuse damage to the central nervous system. In MR examination of the head, 13 patients showed small vascular changes—in 6 patients, vascular changes were found in the visual pathway; in 7 patients, the vascular changes were found in the sensory pathway; and in 5 patients, the vascular changes were found to occur in the bridge. Only one patient with small vasculopathic lesions had an autoimmune disorder—rheumatologic arthritis.

In all patients, ophthalmologic and laryngologic examinations excluded diseases of the visual system (including optic neuritis) and the auditory-vestibular system. Steroid therapy was required in 27 patients, and another immunosuppressive treatment (azathioprine) was required in 8 patients.

## 3. Methods

Evoked potential testing was performed using a Viking Quest apparatus. The procedures were performed according to the guidelines of the International Federation of Clinical Neurophysiology (IFCN) and the American Society of Electroencephalography. Potentials were recorded at a fixed time of day (after breakfast, in the morning). The test was performed on patients in a quiet and darkened room with a constant temperature of 22–24 degrees Celsius. Surface Ag/AgCl cup electrodes of 10 mm diameter from Nicolet Instrument Corporation were used, and these were placed on the scalp according to the international 10–20 scheme and attached with Ten20 Conductive adhesive paste from D.O. Weaver and Co.

VEP, BAEP, and SEP were examined. The stimulus of a specific modality was repeated many times, and using amplification and averaging apparatus, the summed response was selected from the background noise of spontaneous bioelectrical activity and was recorded as a characteristic curve. Analysis of the evoked response included the identification of its individual components (corresponding to the CNS structures in which they are generated), measurement of latency (the time between the stimulus onset and response), and amplitude. The stimulus to obtain the VEP was a reversible checkerboard pattern. The latency and amplitude of the P100 waveform were analyzed. In the BAEP study, the stimulus was a “click”. The auditory brainstem response was evaluated and consisted of five waves appearing over a period of approximately 8 ms, which were assigned to different sections of the auditory pathway. The presence of waves I, II, III, IV, and V, their latencies, and the I–III, III–V, and I–V interlatencies were analyzed. The SEPs were evoked by electrical stimuli of the median nerve and were recorded along the sensory pathway—at the level of the nerve plexus (at Erb’s point), the spinal cord (in the cervical section), and from the cranial surface in the parietal region. The responses were extracted, and their characteristic components were interpreted. The latencies of the SEP components were analyzed: peripheral—N9 and P10, brainstem—N13 and P16, cortical—N20 and P22, and inter-peak interlatency N20—N13, i.e., central conduction time TT. The amplitudes of N9/P10, N13/P16, and N20/P22 were also evaluated.

The study was approved by the Bioethics Committee of Wrocław Medical University (KB-357/2010) and was also supported by Wrocław Medical University SUB.C.220.19.056.

## 4. Statistical Analysis

Statistical analyses were performed in STATISTICA 12.0 with a significance level of alpha = 0.05 for all of the performed tests. Norm values were determined based on the results of the control group for VEP, BAEP, and SEP data. Values within the range (mean − 3 − SD; mean + 3 − SD) were considered the norm according to the three-sigma rule (Three-sigma rule. Encyclopedia of Mathematics. URL: http://encyclopediaofmath.org/index.php?title=Three-sigma_rule&oldid=43551, accessed on 10 August 2021).

The mean values of the individual parameters were compared both between patients and the control group and within the patient group using Student’s *t* test (with normal distribution of variables and homogeneity of variance) and Mann–Whitney’s U test—when variables were not normally distributed. The normality of the distributions was assessed using the Shapiro–Wilk test.

## 5. Results

### 5.1. Evoked Potentials Parameters

Compared to the controls, myasthenia gravis patients showed a significantly longer latency of the P100 VEP waveform (Table 1).

When compared to the controls, the myasthenia gravis group showed statistically longer latencies of the III and V BAEP waves, reduced amplitude of the I and V, and prolonged III–V and I–V interlatency (Table 2).

Myasthenia gravis patients showed significantly longer latencies of N20, P22, N13, P16, N9, and P10 and a decreased amplitude of the N9/P10 somatosensory evoked potentials (Table 3). The mean height of the patients with myasthenia gravis was 172 cm ± 14 cm, and the mean height in the control group 170 cm ± 15 cm (*p* = 0.12).

### 5.2. Correlation of Evoked Potentials Parameters with the Clinical Course of Myasthenia Gravis

Compared to the subgroup of patients with ocular myasthenia gravis, patients with generalized myasthenia gravis showed significantly prolonged latencies of wave III (4.00 ± 0.3 vs. 3.82 ± 0.25; *p*-value 0.045), I–III (2.31 ± 0.27 vs. 2.06 ± 0.16; *p*-value < 0.00001), and I–V (4.30 ± 0.29 vs. 4.08 ± 0.23; *p*-value 0.010) BAEP. Patients with generalized myasthenia gravis also showed a significantly lower amplitude of N13/P16 (0.97 ± 0.64 vs. 1.59 ± 1.2; *p*-value 0.029) and N9/P10 (1.26 ± 1.12 vs. 3.19 ± 2.07; *p*-value 0.000) SEP. There were no statistically significant differences in other parameters of BAEP, SEP, and VEP dependent on the type of myasthenia gravis (generalized/ocular form).

According to the duration of the disease, patients were divided into two subgroups—one with a disease duration of less than 10 years (*n* = 26) and the other with a disease duration of longer than 10 years (*n* = 16). Patients with a disease duration of over 10 years had a significantly lower N9/P10 amplitude (1.35 ± 1.15 vs. 2.54 ± 2.06; *p*-value 0.041) of SEP. Disease duration did not influence the level of the VEP and BAEP parameters.

Patients with autoimmune co-morbidities showed a significantly shorter latency of wave I BAEP (1.75 ± 0.17 vs. 1.67 ± 0.07; *p*-value 0.0002) and a higher amplitude of N9/P10 SEP (2.86 ± 1.2 vs. 1.78 ± 1.98; *p*-value 0.005). There were no significant differences in the VEP parameters dependent on the coexistence of autoimmune diseases.

In the group of patients with vascular lesions on an MR examination compared to the control group, we found significantly longer P100 VEP latency (114.70 ± 8.67 vs. 99.72 ± 3.78; *p* < 0.00001), longer V-wave BAEP latency (5.87 ± 0.25; *p* = 0.008), lower I-wave BAEP amplitude (0.18 ± 0.07 vs. 0.30 ± 0.09; *p* = 0.000), longer I–V BAEP interlatency (4.16 ± 0.23 vs. 3.98 ± 0.19; *p* = 0.038), higher I/V BAEP amplitude (2.72 ± 1.25 vs. 1.61 ± 0.55; *p* = 0.000), and lower N9/P10 SEP amplitude (1.77 ± 1.94 vs2.84 ± 1.89; *p* = 0.041). When comparing the group of patients with MR vascular lesions and the group of patients without MR vascular lesions, significantly shorter III–V BAEP interlatency, shorter N9, P10, N13, P16 waveform latencies, and shorter N13-N9 SEP interlatency were demonstrated in patients with MR vascular lesions (Table 4 and Table 5). There were no statistically significant differences for the VEP parameters in the two patient subgroups.

## 6. Discussion

Previous studies have not clearly confirmed that myasthenia gravis, as an autoimmune disease with involvement of the neuromuscular junction, may involve the entire nervous system, either the central nervous system (CNS), the peripheral nervous system, or the autonomic nervous system. Patients with MG have shown to have headaches, sensory disturbances, dysautonomia, depression, anxiety disorders, sleep disorders, verbal memory disorders, and epilepsy [6,7,8,9,10,11,12]. Sporadic symptoms of myasthenia gravis may also include reversible damage to the pyramidal tract, pseudointernuclear ophthalmoplegia, and neuritis optica [13,14,15]. A multifactorial aetiology of CNS disorders in myasthenia gravis has been hypothesized [2,8,9,12,16]. Abnormalities of the cholinergic system, the influence of non-specific immune factors on the CNS, the lack of protective factors, the type of treatment administered as well as the influence on some symptoms of low patient mood have been considered [16,17].

In scientific literature, there are also case reports of myasthenia gravis coexisting with other diseases associated with CNS pathology [13,14,15,17]. Myasthenia gravis has been reported to be associated with CNS autoimmune diseases, including multiple sclerosis, neuromyelitis optica spectrum disorder (NMOSD), anti-N-methyl-d-aspartate receptor (NMDAR) encephalitis, and Morvan syndrome [17,18]. In addition, patients with MG may develop autoimmune diseases such as thyroid ophthalmopathy, Hashimoto thyroiditis, Graves’ disease, rheumatoid arthritis, systemic lupus erythematosus, Sjögren’s disease, Behçeti disease, polymyositis, Guillain–Barre syndrome, and pernicious anaemia [10,19,20,21]. The immunobiological mechanism of the co-occurrence of MG and other autoimmune disorders is not fully understood. In NMOSD, some studies have shown that aquaporin 4 water channels (AQP4) are expressed at the neuromuscular junction in thymocytes of patients with MG and thymoma [22,23,24]. Additionally, Vaknin-Dembinsky et al. [23] discovered the presence of anti-AQP4 antibodies in MG with marked CNS involvement (the presence of significant hyperreflexia and soft pyramidal signs). They speculated that CNS involvement in MG is more common than shown in previous studies and is primarily expressed by involvement of the pyramidal tract, with associated visual pathway damage and white matter changes in MR images, which may resemble neuromyelitis optica spectrum disorder (NMOSD). Thymomas are more frequently found in MG patients with CNS involvement [1,4,10]. Thymic abnormalities associated with MG predispose patients to the production of specific autoantibodies directed against CNS structures. However, according to other publications, the relative increase in the prevalence of patients with both AQP4-NMOSD and AChR-MG suggests that these patients have a predisposition to autoimmunity, but the dynamics of the individual diseases remain unchanged [22].

Observations indicate that patients with different subtypes of myasthenia gravis, associated with the presence of different antibodies—AChR, MuSK, or against lipoprotein receptor-related protein 4 (LRP4)—present similar clinical symptoms, regardless of the mechanism of immunopathology [25]. In the subtype with anti-AChR antibodies, which belong to the IgG1 subclass, long-lived plasma cells play a predominant role in the pathogenesis. The similarity underlying the immunopathology of AChR-antibody-positive MG and NMO is highlighted [22,23]. In contrast, MuSK-antibody-positive MG is caused by autoantibody production by short-lived plasmablasts. These autoantibodies mainly belong to the IgG4 subclass, which can undergo Fab-arm exchange (FAE), a process unique to this subclass. In the FAE process, IgG4 molecules can dissociate into two halves and recombine with other half IgG4 molecules resulting in bispecific antibodies [25,26,27,28].

Immunological studies, supported by animal experiments, indicate that the presence of muscle anti-AChR antibodies in cerebrospinal fluid (CSF) is not evidence of CNS dysfunction in myasthenia gravis because these antibodies have no affinity for AChR present in the brain [6]. In most MG patients, no IgG oligoclonal bands are found in the CSF (28). However, Zhang et al. [10] showed an increased ratio of AChRAb in CSF to AChRAb in serum (*p* < 0.05) in MG patients with co-present pyramidal symptoms (without known causes of pyramidal tract damage). They hypothesized that pyramidal symptoms are caused by the intrathecal synthesis of IgG antibodies associated with the pyramidal tract.

Reports on the bioelectrical assessment of CNS function in MG are not numerous. We evaluated ocular vestibular evoked myogenci potentials, which analyse the function of vestibular nerves, brainstem vestibular nuclei, and their projection to the cervical cord alpha motoneurons with reception on the sternocleidomastoid muscles. Statistical differences in these potentials have been demonstrated between myasthenia gravis patients that are uncontrolled and controlled by treatment and between ocular and generalized types of myasthenia gravis [29]. Other studies have reported changes in VEP and BAEP in MG [13,30,31,32].

Thus, there is no certain evidence indicating primary CNS dysfunction in MG, but secondary CNS dysfunction cannot be excluded [6]. The literature also highlights the effects of long-term steroid therapy on some structures of the eye and visual pathway. Chronic steroid treatment in MG patients may cause the development of glaucoma with optic nerve damage, posterior polar cataracts, and central serous chorioretinopathy [19]. In our study, we demonstrated a significantly longer latency of the P100 VEP waveform in patients with myasthenia gravis, although none of them had post-steroidal ocular complications. Similar results were obtained by Fotiou et al. [30], who found significantly prolonged P100 latency and significantly lower P100 PR-VEP amplitude in patients with recently diagnosed myasthenia gravis. After the inclusion of pyridostigmine treatment and a good therapeutic effect, PR-VEP follow-up studies showed an increase in P100 amplitude in the patient group. These results were interpreted by the authors as neurophysiological evidence of impaired cholinergic transmission in CNS in patients with MG and suggested that PR-VEP may be a non-invasive method to study the effects of MG on the CNS and a procedure that can monitor the effectiveness of pyridostigmine treatment.

In our BAEP study, we found a significantly longer latency of refraction III and V, longer interlatency of III–V and I–V, and lower amplitude of I, V, and I/V in MG patients compared to healthy subjects. The prolongation of interlatency between components III and V and I–V of the evoked response shown in our study is a sensitive indicator of impaired stimuli conduction in the auditory pathway within the brainstem. In the case reports presented in the literature of MG patients with nervous system involvement, the BAEP parameters are normal [14]. Only Jech et al. [32] performed BAEP in 40 MG patients with forms II and III according to the Ossserman classification. In the patient group, there was a significant decrease in the amplitude of wave I and an increase in the amplitude of wave III in the fourth series compared to the first series in the patient group. The control group of healthy subjects showed a decrease in the wave III amplitude as a normal habituation process. It was suggested that the increase in wave III amplitude during prolonged stimulation in MG patients may illustrate an amplifying mechanism of brainstem neuron responses, particularly at the level of the superior olive complex of the brainstem. The authors base their hypothesis on the concept of central cholinergic dysfunction in myasthenia gravis, suggesting two mechanisms for the increase in wave III amplitude.

In the available literature, we did not find any study analysing the SEP parameters in myasthenia gravis patients. In a sporadic case of optic neuritis in a myasthenic patient, BAEP and SEP were normal [14]. In our study, we demonstrated a significantly prolonged latency of all of the SEP components obtained, with median nerve stimulation and a lower amplitude of N9/P10 in the patient group. These changes may be due to the slower conduction of sensory stimuli in the peripheral neurons. Vernino et al. [11] described 7 patients with myasthenia gravis and subacute autonomic neuropathy—all showed antibodies to muscle AChR, and three (all of whom had thymoma) had antibodies to ganglionic neuronal AChR [21,33,34,35]. The coexistence of MG with neuropathy and neuromyotonia has also been reported [36] as well as with subacute motor neuropathy, in which antibodies against the axon, possibly against neurofilament or tubulin components, have been suggested [37].

SEP results do not indicate damage to CNS function. This is supported by the lack of significant differences between the patients and the control group in the interlatency N13-N9 (assessment of conduction between the plexus and the lower part of the medulla oblongata) and N20-N13 (assessment of conduction within the medial ribbon and the thalamocortical pathway of the opposite hemisphere). However, the demonstrated BAEP changes (prolongation of III–V and I–V interlatencies) indicate the involvement of central afferent pathways in MG.

Comparing the generalized form with the ocular form of myasthenia gravis, in our study, we found changes in the generalized form, indicating disorders within the brainstem (BAEP and SEP changes) and in the peripheral nervous system (SEP changes). Changes in the N9/P10 amplitude of SEP suggesting peripheral nervous system abnormalities were also demonstrated in patients with longer disease duration.

Evoked potential abnormalities are not a direct reflection of changes in individual CNS structures and may be modified by a focus on brain damage that is distant in location from the neural pathway stimulated by a given stimulus modality. Inhibition of the sensory activity of the cerebral cortex due to focal damage causes degenerative changes in the thalamic neurons [31]. The longer latencies of the evoked potentials that were observed in our patients may be due to the influence of myasthenia gravis as a generalized autoimmune process on the CNS.

The limitations of our study include conducting the study in only one time point in myasthenia gravis patients, which is known to have a fluctuating clinical course. Another limitation is the relatively small group of patients and a slightly younger control group in relation to the study group—both groups were not well-matched for gender and age. However, this is a preliminary report, and we plan to continue our research in a long-term follow-up.

## 7. Conclusions

The changes in evoked potentials that we demonstrated indicate that MG is not only a disease of the neuromuscular junction, but also involves afferent pathways of the peripheral and central nervous system.

## Figures and Tables

**Table 1:** VEP parameters in myasthenia gravis patients and in the control group.

VEP		Study Group (*n* = 42) Mean ± SD	Control Group (*n* = 50) Mean ± SD	*p*-Value *
Latency (ms)	N75	79.1 ± 16.1	69.8 ± 5.2	0.0007
	P100	113.9 ± 13.9	99.7 ± 3.8	<0.0001
	N145	156.0 ± 18.6	142.0 ± 10.2	0.0004
Amplitude (μV)	P100/N145	9.45 ± 4.8	10.1 ± 3.6	0.2417

VEP—visual evoked potentials, uV—microvolts, ms—millisecond, SD—standard deviation. * with Bonferroni correction (alfa = 0.0125).

**Table 2:** BAEP parameters in myasthenia gravis patients and in the control group.

BAEP		Study Group (*n* = 24) Mean ± SD	Control Group (*n* = 50) Mean ± SD	*p*-Value *
Latency (ms)	I	1.72 ± 0.15	1.65 ± 0.12	0.056
	III	3.92 ± 0.29	3.81 ± 0.17	0.015
	V	5.93 ± 0.32	5.64 ± 0.16	<0.0001
Amplitude (µV)	I	0.18 ± 0.08	0.31 ± 0.10	<0.00001
	V	0.35 ± 0.12	0.42 ± 0.11	<0.003
Interlatency (ms)	I–III	2.20 ± 0.25	2.12 ± 0.12	0.919
	III–V	2.00 ± 0.12	1.85 ± 0.14	<0.0001
	I–V	4.20 ± 0.28	4.01 ± 0.15	<0.001

BAEP—brainstem auditory evoked potentials, uV—microvolts, ms—millisecond, SD—standard deviation. * with Bonferroni correction (alfa = 0.0056).

**Table 3:** SEP parameters in myasthenia gravis patients and in the control group.

SEP		Study Group (*n* = 42) Mean ± SD	Control Group (*n* = 50) Mean ± SD	*p*-Value *
Latency (ms)	N9	10.66 ± 1.59	9.77 ± 0.90	0.007
	P10	12.47 ± 2.48	11.32 ± 1.01	0.001
	N13	14.36 ± 2.44	13.14 ± 1.12	0.015
	P16	17.47 ± 2.48	16.22 ± 1.11	0.007
	N20	20.98 ± 2.85	19.27 ± 1.08	0.003
	P22	24.10 ± 3.48	22.25 ± 1.63	0.006
Interlatency (ms)	TT (N20–N13)	6.62 ± 1.25	6.13 ± 0.84	0.103
	N13-N9	3.70 ± 1.11	3.41 ± 0.79	0.294
Amplitude (μV)	N9/P10	2.09 ± 1.85	2.85 ± 1.91	0.045
	N13/P16	1.24 ± 0.96	1.08 ± 0.32	0.684
	N20/P22	1.15 ± 0.97	0.96 ± 0.50	0.760

SEP—somotosensory evoked potential, TT—transit time, uV—microvolts, ms—millisecond, SD—standard deviation. * with Bonferroni correction (alfa = 0.0046).

**Table 4:** BAEP values in patients with and without vascular lesions on MR imaging.

BAEP		Absence of Ischemic Lesions *n* = 29	Presence of Ischemic Lesions *n* = 13	*p*-Value
		Mean ± SD	Mean ± SD	
Latency (ms)	I	1.75 ± 0.19	1.70 ± 0.16	0.369
	III	3.90 ± 0.22	3.92 ± 0.26	0.925
	V	5.99 ± 0.28	5.87 ± 0.25	0.310
Amplitude (µV)	I	0.14 ± 0.08	0.18 ± 0.07	0.337
	V	0.28 ± 0.11	0.37 ± 0.11	0.086
Interlatency (ms)	I–III	2.15 ± 0.16	2.21 ± 0.24	0.532
	III–V	2.09 ± 0.10	1.95 ± 0.16	0.041
	I–V	4.24 ± 0.21	4.16 ± 0.23	0.459

BAEP—brainstem auditory evoked potentials, uV—microvolts, ms—millisecond, SD—standard deviation.

**Table 5:** SEP values in patients with and without vascular lesions on MR imaging.

SEP		Absence of Ischemic Lesions *n* = 29	Presence of Ischemic Lesions *n* = 13	*p*-Value
		Mean ± SD	Mean ± SD	
Latency (ms)	N9	11.93 ± 2.69	10.11 ± 1.23	0.022
	P10	13.21 ± 1.31	12.00 ± 2.16	0.025
	N13	16.60 ± 4.23	13.51 ± 1.49	0.022
	P16	19.73 ± 4.15	16.70 ± 1.50	0.022
	N20	23.27 ± 4.83	20.23 ± 2.02	0.079
	P22	26.39 ± 5.72	23.77 ± 2.93	0.270
Interlatency (ms)	N20-N13 (TT)	6.67 ± 1.25	6.72 ± 1.58	0.903
	N13-N9	4.67 ± 1.80	3.40 ± 0.80	0.025
Amplitude (μV)	N9/P10	1.98 ± 1.57	1.77 ± 1.94	0.713
	N13/P16	1.39 ± 0.93	1.22 ± 1.32	0.206
	N20/P22	0.90 ± 0.48	1.37 ± 0.91	0.186

SEP—somotosensory evoked potential, TT—transit time, uV—microvolts, ms—millisecond, SD—standard deviation.

## Data Availability

The data presented in this study are available upon request from the corresponding author. The data are not publicly available.

## Abbreviations

AChRAb	anti-acetylcholine receptor antibodies
AQP4	aquaporin 4
BAEP	brainstem auditory evoked potentials
CIDP	chronic inflammatory demyelinating polyneuropathy
CNS	central nervous system
EP	evoked potentials
IFCN	International Federation of Clinical Neurophysiology
MG	myasthenia gravis
MRI	magnetic resonance imaging
MuSK	muscle-specific tyrosine kinase
NMDAR	anti-N-methyl-d-aspartate receptor
NMOSD	neuromyelitis optica spectrum disorders
SEP	somatosensory evoked potentials
VEP	visual evoked potentials
